# Universal Society of Ophthalamology

**Published:** 1862-08

**Authors:** 


UNIVERSAL SOCIETY OF OPHTHALAMOLOGY.—The object of this Society is to advance the science of Ophthalomology by yearly meetings, establishing a pathological collection, and publishing transactions. It is to change its seat of action yearly from one to another of the great scientific centres of the world. Eleven such centres, all of them European, have so far been selected. The most celebrated names figure in its list of committees, such as Sichel, Desmarries, Von Græfe, Bowman, Arlt, Mackenzie, Wilde, etc., etc. We learn that Drs. Valentine Mott and Julius Homberger, of this city, and Drs. Pancoast, Hayes and Little, of Philadelphia, compose the American Committees.

The first meeting of the Society will be held at Paris, between September

30th and October 3d, of this year; and the Central (Parisian) Committee, has issued a request to the government of all civilized nations to send delegates to its sessions, We presume the American Committees will soon officially invite all parties interested to participate.

To the Medical Profession of the United States.—The object of the Universal Society of Ophthalmology is known to you, and we hope its foundation will mark the present year in the annals of Medical Science.—We are fully satisfied that the Society will take, from its first meeting, the position which it has a right to ask among scientific bodies. We believe it is now the proper moment to solicit your help and sympathy.

"We invite you to associate yourself with the Society, which will meet for the first time, from the 30th of September to the 3d of October, 1862, in Paris.

"Your desire to be enrolled on its list of membership is requested to be made known to one of the undersigned, who will forward it to the Central Committee.

"*The Committee of the State of New York:*

"Valentine Mott, M. D., 1 Gramercy Park,

"Julius Homberger, M. D., 24 West Twelfth St.

"New York, May 20th, 1862."

